# The Role of Interoceptive Attention and Appraisal in Interoceptive Regulation

**DOI:** 10.3389/fpsyg.2021.714641

**Published:** 2021-10-25

**Authors:** Vrutti Joshi, Pierluigi Graziani, Jonathan Del-Monte

**Affiliations:** ^1^Department of Psychology, University of Nîmes, Nîmes, France; ^2^Social Psychology Laboratory EA 849, Aix-Marseille University, Marseille, France

**Keywords:** interoception, interoceptive attention, interoceptive awareness, psychopathology, interoceptive regulation

## Abstract

Interoception, i.e., the processing and integration of sensory information has gained research interest due to its relevance in the psychopathological context. In the present review, we focus upon interoceptive regulation or one’s capacity to match bodily signals to his/her desired state by altering the signal or the desired state. More specifically, we discuss attention toward and appraisal of interoceptive stimuli as regulatory mechanisms of interoception. We review findings in the emerging research area of interoceptive attention. Studies suggest that the quality of attention and the nature of appraisal regarding interoceptive information influence interoceptive regulation and subsequent adaptive or maladaptive behavioral strategies among healthy controls as well as clinical populations. We discuss the clinical implications and the need to promote further research as well as to target interoceptive attention and appraisal mechanisms in psychotherapy.

## Introduction

Interoception can be defined as the mechanism by which the nervous system processes and integrates bodily information (Khalsa and Lapidus, 2016). It has been suggested to link underlying interoceptive processes to psychological disorders, following a trans-diagnostic and a dimensional approach (Khalsa et al., 2018). Studies have focused on the possible implications of atypically high or low interoceptive abilities in the psychopathological context (Murphy et al., 2017). However, very few studies elaborate on the facet of interoceptive attention. The narrow definition of interoception refers to the integration of physical sensations and its precision (Khoury et al., 2018). Whereas the broad definition of interoception highlights the role of the psychological context in which the individual perceives and processes stimuli, one’s quality of attention and trust toward these bodily signals and his ability to listen to physical sensations to regulate decisions and behaviors. The attentional and appraisal processes, i.e., the processes which enable us to notice, categorize, interpret, and respond to our physical sensations would hence comprise the regulatory aspects of interoception (Bornemann et al., 2015; Mehling, 2016; Khoury et al., 2018). Rather than a categorical “interoceptive dysfunction” discussed in previous studies, we highlight evidence for interoceptive regulation as well as attention and appraisal as regulatory aspects of interoception, given that the notion that interoceptive processes are modifiable *via* treatment is essential in the psychopathological field (Farb et al., 2015; Khoury et al., 2018).

## Interoception: Dimensions and Measurement

As per Garfinkel et al. (2015), interoception comprises: (a) objective performance on interoceptive measures (interoceptive accuracy), (b) beliefs about one’s interoceptive capacities (interceptive sensibility), (c) the correspondence between objective interoceptive accuracy and subjective sensibility (metacognitive interoceptive awareness). Heartbeat detection tasks are the most utilized methods to measure interoceptive accuracy; individuals are required to count the number of heartbeats perceived in a specific timeframe (“Heartbeat tracking,” Schandry, 1981) or report if their heartbeats are synchronous with external stimuli (“Heartbeat discrimination,” Katkin et al., 1983). Self-report questionnaires are employed to evaluate the subjective components of interoception (e.g., Body Perception Questionnaire, Porges, 1993; Interoceptive Accuracy Scale, Murphy et al., 2020, etc.) (Garfinkel et al., 2015; Murphy et al., 2019).

Murphy et al. (2019) proposed the 2 × 2 model which highlights the measurement target (accuracy versus attention) as well as the measurement method (objective measure/self-report). Thus, the 2 × 2 model underlines the importance of evaluating individual differences in the degree up to which individuals employ their attention to interoceptive stimuli and use bodily information in their daily lives. So far, the Multidimensional Assessment of Interoceptive Awareness (MAIA) (Mehling et al., 2012) is employed as a self-report tool to measure interoceptive sensibility or attention, differentiating adaptive, and maladaptive attentional styles. However, precise measurement of interoception would require tapping a discernible bodily channel to measure interoceptive attention. The Breath Detection Task (Wang et al., 2019) was devised to engage interoceptive attention to one’s breathing rhythm to evaluate underlying neural processes. Similarly, the Visceral Interoceptive Awareness (Stewart et al., 2020) and the heartbeat attention task (Petzschner et al., 2019) evaluate interoceptive attention by measuring specific visceral channels and underlying neural activity. In the present mini review, we aim to highlight interoceptive attention and appraisal as factors influencing the interoceptive regulation process, subsequent adaptive or maladaptive cognitive, emotional, and behavioral regulation strategies. Understanding the role of specific interoceptive dimensions in interoceptive regulation will promote interventions to target factors contributing to interoceptive dysregulation.

## Selection Method

References were identified through a search of the keywords “interoceptive regulation” and “interoceptive attention” on PubMed. Studies were selected based on their pertinence in the present review and relevant references of the selected articles were searched as well. The present review only included articles published in English between 2015 and 2021.

## Interoceptive Regulation

Interoception is a sequential process requiring an interaction between perceiving bodily states and cognitively appraising these states to facilitate the selection of adequate, regulatory responses. The perceived bodily information is integrated into a conceptual simulation map, i.e., a filtered internal representation of afferent signals. It is assumed that the brain constantly builds a model of incoming sensory information, attributes cause regarding these sensations (inference), actively updates models (learning) according to new bodily information and predicts probable incoming signals (Petzschner et al., 2019). As conceptualized by Farb et al. (2015), interoceptive regulation refers to an individual’s capacity to match interoceptive signals to his desired state, by altering the signal or the desired state. Learning or updating beliefs is induced by the gap between predicted and actual incoming sensory information, operationalized as prediction errors. Attention toward and away from interoceptive stimuli thus plays an essential role in this model (Petzschner et al., 2019). Regulatory motivation is largely impacted by the extent to which unpredicted physical sensations are regarded as acceptable rather than menacing and devious from one’s anticipated bodily state (Farb et al., 2015).

As explained by Khoury et al. (2018), interoception, i.e., the perception of bodily sensations, and the resulting attentional and appraisal processes in response to stimuli are determined by trait-like tendencies toward interoceptive experiences such as accuracy, attentional tendency, confidence, etc. Taken together, the conscious or unconscious state and trait-like interoceptive facets promote regulatory interoceptive strategies much alike emotional regulation strategies. In other words, the relationship between interoceptive perception and resulting interoceptive regulation, at a given moment, is impacted by the degree of attention and appraisal toward stimuli in that given moment, which itself is determined by the individual’s general tendency to pay attention to, trust, and accurately perceive his bodily sensations. Finally, interoceptive regulation further impacts the perceptual, attentional, and appraisal mechanisms in response to interoceptive cues, in a feedback loop (Farb et al., 2015; Khoury et al., 2018).

## Targeting Interoceptive Regulation in Psychopathology

Indeed, psychiatric conditions are characterized by impairment in interoceptive accuracy, awareness, sensibility, and response to interoceptive signals (Paulus and Stein, 2010; Ardizzi et al., 2016; Sönmez et al., 2017; Jenkinson et al., 2018; Khalsa et al., 2018; Löffler et al., 2018; Bragdon et al., 2021). Accurate perception of interoceptive signals (interoceptive accuracy) is not only essential for their regulation but also implied in psychopathology alongside interoceptive sensibility and awareness (Murphy et al., 2017). One approach to psychopathology was devised based on the brain’s way to compute and integrate information from the body’s inner and outer worlds. As per Paulus et al.’s (2019) active inference approach to psychopathology, mood, and anxiety related changes would be explained by the brain’s biased beliefs or expectations of what might happen as opposed to what really occurs, leading to discrepancies or error signals. Psychopathology would thus originate from one’s inflexible internal representations of a constantly shifting external world. Individuals with “hyperprecise” priors, i.e., strong beliefs regarding one’s internal model as being correct, would not easily generate alternate models accounting for incoming afferent signals (Paulus et al., 2019). Indeed, worry and rumination may further reinforce hyperprecise priors by hindering correction despite sensory evidence (Paulus and Stein, 2006).

Individuals with panic disorder may portray hyperprecise priors and an inability to modify the same despite incoming sensory evidence. This is illustrated by an individual sensing his heartbeat and fearing a heart attack while experiencing a panic attack. The authors theorize that depression may be characterized by persistent error signals originating from the discrepancy between prior beliefs and incoming interoceptive information. Context rigidity or the inability to adapt one’s internal representation to varying contexts may also characterize interoceptive difficulties in depression. Hyperprecise errors which are context-specific (e.g., hunger-related) may generate strong anticipations about bodily sensations. The hallmark symptom of starvation of anorexia nervosa, however, would be a behavioral response to hyperprecise errors to avoid real-time visceral sensations. Moreover, the authors suggest that there may be a common link between individuals with panic disorder and somatic symptom disorders, given the comorbidity, aversive expectations, heightened attention, and negative interpretations about interoceptive cues, marked with a difficulty to update one’s priors (Paulus et al., 2019).

One primary approach to target interoceptive dysregulation involves active inference (Friston et al., 2009) techniques such as shaping or restoring one’s interoceptive sensations to match his anticipated or desired bodily state (Farb et al., 2015). For an individual suffering from panic disorder, distracting one’s attention away from the heartbeat, active methods of decreasing symptoms such as medication or cognitive re-appraisal to re-interpret the significance of the increase in heart rate are relevant and frequent treatment goals (Khoury et al., 2018). The second method to target interoceptive regulation is *via* “perceptual inference” techniques wherein the anticipated simulation map is updated to be more representative of current physical sensations. Perceptual inference involves shifting one’s attentional focus to the present state rather than prior beliefs. Adequately implementing these strategies may allow the individual to update his expectations or develop context-specific insight into his bodily state, thus facilitating opportunities for regulation. Exposure and mindfulness-based techniques may be applied to develop acceptance rather than the modification of the physiological anxiety manifestations (Farb et al., 2015; Khoury et al., 2018).

Paulus et al. (2019) provide a theoretical active inference approach to psychopathology and highlight the need to investigate the same in their experimental framework. In the next section, we review experimental findings on interoceptive attention and the appraisal of bodily information that highlight their role as a gateway to interoceptive regulation.

## Recent Findings Supporting the Regulatory Role of Interoceptive Attention and Appraisal

Interoceptive attention can be defined as the conscious focus and awareness of bodily sensations. As shown by Wang et al. (2019) (Table 1), the anterior insular cortex (AIC) was seen to play a role in interoceptive attention, individual differences in interoceptive accuracy and the predictive coding of breathing-based interoception. Murphy et al. (2020) empirically demonstrated the independence of subjective interoceptive accuracy from subjective interoceptive attention and highlighted the necessity to develop refined measures of interoceptive dimensions. Furthermore, Petzschner et al. (2019) demonstrated that attentional focus modulates the heartbeat evoked potential (HEP) which confirms the claim that the HEP is a neural correlate of interoceptive prediction error information. Interoceptive attention thus increases the precision of sensory signals, consequently increasing the intensity of prediction errors related to attention on exteroceptive cues; exteroceptive attention downregulates the salience of bodily information. In other words, the results highlight the essential role of interoceptive attention, showing that the modulation of attention toward and away from the heart is essential to interoceptive processing as reflected in the HEP amplitude.

**TABLE 1:** Studies assessing interceptive attention, appraisal, and interoceptive regulation.

	Authors	Interoception measurement (facet)	Sample	HC	Results
1	Wang et al., 2019	Breath Detection Task (interoceptive attention, interoceptive accuracy), Body Perception Questionnaire (interoceptive sensibility)	Lesion study *N* = 6	Sample 1 (fMRI study) *N* = 44; Sample 2 (fMRI study) *N* = 28; Lesion study Healthy controls *N* = 12	Association of anterior insular cortex (AIC) with interoceptive attention, AIC activation was seen to predict individual differences in interoceptive accuracy.
2	Wiersema and Godefroid, 2018	Mental tracking method (objective accuracy), Body Perception Questionnaire (interoceptive awareness)	Adults with ADHD *N* = 24	*N* = 23	No significant difference in objective and subjective measures of interoceptive awareness between patients and healthy controls.
3	Stewart et al., 2020	Multidimensional Assessment of Interoceptive Awareness, Visceral Interoceptive Aware-ness (VIA) task (interoceptive attention)	Current Stimulant Use Disorder (SUD) *N* = 40, current Opioid Use Disorder (OUD) *N* = 20	*N* = 30	Patients with SUD experience a greater intensity of heartbeat sensations. Blunted insula responses were observed for stomach sensations in OUD and SUD.
4	Wu et al., 2019	Interoceptive attention task (fMRI)	General population *N* = 127		The COTC network is implied in excessive attention to interoceptive cues whereas the connection between sensorimotor areas and the salience network is more implicated in anxiety.
5	Yao et al., 2018	Heartbeat detection task (interoceptive accuracy)	General population *N* = 83		Intranasal oxytocin decreased interoceptive accuracy when presented with face stimuli, increased right anterior insula activation as well as functional connectivity between anterior and posterior insula.
6	Petzschner et al., 2019	Heartbeat attention task (interoceptive attention), Body Perception Questionnaire (bodily awareness)	General population *N* = 19		Attention modulates heartbeat evoked potential (HEP) amplitude for interoceptive attention.
7	Murphy et al., 2020	Interoceptive Accuracy Scale (self-reported interoceptive accuracy)	6 validation studies of the Interoceptive Accuracy Scale		Results report that subjective interoceptive accuracy and subjective interoceptive attention are distinct constructs.
8	Tan et al., 2018	Heartbeat perception task (interoceptive attention)	General population *n* = 50		Cardiac interoception was seen to be associated to mid, anterior, and posterior insular activation. Anxiety-oriented activation related only to anterior insular activity. Anterior insular activation focused on cardiac interoception is correlated to state and trait anxiety. In addition, mid-insular activation during cardiac attention is associated to interoceptive accuracy, state, and trait anxiety.
9	Todd et al., 2019	Multidimensional Assessment of Interoceptive Awareness (interoceptive awareness)	General population *n* = 265		Four out of five interoceptive awareness facets (Noticing, Trusting, Not-worrying, and Not-distracting) predict at least one body-image facet.
10	Schuette et al., 2020	Multidimensional Assessment of Interoceptive Awareness (interoceptive awareness), heartbeat perception task (interoceptive accuracy)	General population *n* = 95		Noticing bodily sensations is associated to emotion identification, regulation and using adaptive coping styles.
11	Willem et al., 2019	Five Facets Mindfulness Questionnaire (interoceptive awareness), Multidimensional Assessment of Interoceptive Awareness (interoceptive awareness)	Normal weight *N* = 55, Moderately obese *N* = 55, Severely obese *N* = 55		Obese individuals showed higher difficulties in emotion regulation, less interoceptive awareness, less planification strategies, lower emotional awareness, less trust, and observation of bodily cues than normal weight individuals. No significant differences were found among moderately and severely obese subjects.
12	Calì et al., 2015	Multidimensional Assessment of Interoceptive Awareness (interoceptive awareness), heartbeat perception task (interoceptive accuracy)	General population *n* = 321		Results reveal distinction between the constructs of interoceptive accuracy and interoceptive awareness. Emotional susceptibility was seen to be associated to more worry about pain sensations, emotional awareness, lesser trust in bodily information and attention regulation with respect to physical sensations.

*ADHD, Attention deficit hyperactivity disorder; COTC, cingulo-opercular task control.*

With regards to attention as a regulatory aspect of interoception in the psychopathological context, Wiersema and Godefroid (2018) argue that despite interoceptive awareness abilities, individuals with ADHD may not sufficiently use bodily information for self-regulation. Distraction and attenuated attention to bodily information during daily situations may impact awareness and self-regulation. The “attentional switching hypothesis” suggests that challenging social situations may reduce one’s attention toward monitoring his internal states. Thus, consistent with the attentional modulation reflected in the HEP in the previous study discussed above (Petzschner et al., 2019), switching one’s attention between internal (interoceptive) cues and external social cues, along with trust in one’s body signals would facilitate interoceptive abilities as well as social connection (Arnold et al., 2019).

In a recent evaluation of interoceptive attention, Stewart et al. (2020) found that Opioid Use Disorder (OUD) and Stimulant Use Disorder (SUD) patients showed lower self-regulation, body trust, and higher worry about physical sensations compared to controls. SUD patients demonstrated poorer attention regulation of interoceptive cues than controls. Although heartbeat sensations demonstrated higher intensity rates in SUD patients, their valence was unaccounted for. However, the appraisal of sensory effects by the drug users would impact the drug’s reinforcement. Repeated exposure may sensitize the insula by the downregulation of attentional resources allotted to these sensations, causing users to need higher drug doses to maintain their desired bodily/feeling states. Inversely, excessive bodily signal perception would originate from the incapacity to downregulate the importance of sensations in contexts undemanding attention toward the body (Petzschner et al., 2019). Thus, interoceptive attention modulation and the appraisal of sensory experiences in different situations would influence interoceptive regulation by updating expectations or the desired bodily state itself.

Yao et al. (2018) explored the effects of intranasal oxytocin (OT) on behavioral responses. OT may promote an attentional switch from internal interoceptive cues to socially salient external cues, resulting in decreased interoceptive accuracy. The authors infer that attention failure or impacted interoceptive inference may play a role in autism spectrum disorder (ASD) and schizophrenia due to distorted inferences about bodily states. Trevisan et al. (2021) argued that ASD is characterized by poor interoceptive accuracy and high maladaptive self-reported interoceptive attention related to anxiety-induced somatization. As per the authors, due to the confusion in interoceptive taxonomy, hypervigilance or anxiety-related somatization is often misleadingly considered as representative of superior interoceptive abilities. The authors specify that while interoceptive attention toward physiological sensations is adaptive for anxiety regulation, exaggerated attention to bodily cues may cause higher anxiety.

Amplification of body signals and higher anxiety would be explained by atypical patterns in specific brain areas, as demonstrated by Wu et al. (2019). The connectivity in the cingulo-opercular task control (COTC) network (consisting of the dorsal anterior insula and the ventral anterior cingulate cortex) may indicate exaggerated attention toward interoceptive signals. Tan et al. (2018) found that anterior insular activity indicated attention to bodily information; anterior insular activity was associated to state and trait anxiety levels. Mehling (2016) assert that merely being aware of one’s bodily sensations without the ability to regulate distress would contribute to anxiety. In their research, individuals manifesting higher anxiety presented lower confidence in interpreting and regulating symptoms of physical arousal. It can thus be inferred that focusing not only on interoceptive attention but also interoceptive regulation techniques can be a key target in research and psychotherapy.

There is a growing need to distinguish interoceptive facets as well as adaptive and maladaptive attentional styles for clinical and research purposes. To illustrate, attentional styles as measured by the MAIA would help promote pain research. In a related study, comparison of MAIA scores between patients experiencing lower back pain and individuals trained in mind-body therapy indicated that patients experiencing pain showed a preference for distraction strategies to cope with pain or discomfort. To the contrary, individuals trained in yoga and/or meditation may have developed a mindful attentional style toward pain (Mehling, 2016). In another study, individuals who scored higher on emotional susceptibility, i.e., the tendency to feel discomforting feelings toward emotional stimuli, showed more worry about pain sensations, higher emotional awareness, less trust toward their bodily information, and less attention to bodily information (Calì et al., 2015).

In a study of body image among adolescents, Todd et al. (2019) concluded that rather than the tendency of noticing interoceptive information, appraisal, and regulation of interoceptive stimuli was more closely associated to body image. Mindfulness-based attention may thus be promising in the development of a positive body image. In the obesity context, Willem et al. (2019) concluded that obese individuals paid significantly less attention to their physical sensations, had greater difficulty in noticing their bodily information, showed lower trust in their physical sensations and the ability to use them to guide behavior, than normal weight individuals. Obese people would pay less attention to their bodily state if they do not regard their bodily information to be trustful and useful, thus leading to emotion regulation difficulties. Indeed, obese people were found to use fewer adaptive cognitive and emotion regulation strategies. In yet another study, the capacity to notice one’s bodily sensations was positively correlated to emotion identification and predicted adaptive coping behaviors. The authors assert that the extent up to which interoception is adaptative would depend upon the quality and degree of attention employed to bodily information (Schuette et al., 2020). Constructive reappraisal of interoceptive information and self-regulation skills may thus promote emotion regulation in individuals presenting a lack of trust in one’s body and coping mechanisms such as depersonalization and distraction (Zucker et al., 2017; Price and Hooven, 2018).

## Discussion

To our knowledge, the role of interoceptive attention and appraisal in the regulation of interoceptive stimuli is relatively unexplored. Interoception is a complex, multifaceted process. Further research highlighting the mechanisms of specific variables contributing to interoceptive regulation will promote targeting interoceptive dysregulation in the psychopathological context *via* adapted psychotherapy protocols.

Further research exploring interoceptive attention and regulation across various psychological disorders is needed. Recent studies have targeted, *via* interoceptive training practices, the improvement of interoceptive accuracy as measured by the heartbeat detection task but results have been inconsistent. One plausible explanation may be that contemplative practices may only impact specific interoceptive dimensions (see Gibson, 2019). Studies found significant improvements in the subjective self-regulatory, attentional dimensions of interoception (Bornemann et al., 2015; De Jong et al., 2016; Fissler et al., 2016). Therapeutic interventions such as Somatic Experiencing (Payne et al., 2015), Mindful-Awareness in Body-Oriented Therapy or MABT (Price and Hooven, 2018), or body-based contemplative practices such as Qigong and Tai-Chi for mood regulation (Yeung et al., 2018) target attention to interoceptive stimuli. Zucker et al. (2017) demonstrated the efficacy of an acceptance based interoceptive exposure protocol for functional abdominal pain among children inspired from the interoceptive exposure protocols for panic disorder. Behavioral exposure to body signals would provide individuals with the opportunity to interpret bodily signals more accurately rather than associate them to menace, reduce behavior avoidance and generalize adaptive rather than fear-based reactions. Hence, this approach would promote curiosity toward one’s bodily information as a resource of useful information to guide behavior. In their comprehensive review of interoceptive interventions among patients with psychiatric conditions, Khoury et al. (2018) highlight the importance of CBT interventions for interoceptive regulation *via* active inference techniques such as cognitive restructuring and perceptual inference strategies that promote acceptance, such as exposure, mindfulness, and self-observation.

## Conclusion

To conclude, future research outlining attention to interoceptive stimuli and the attribution of a positive or a negative valence seems promising to tackle interoceptive dysregulation in psychopathology. More accessible interoceptive attention tasks will help promote research in the mental health arena. Psychotherapeutic interventions focused upon the modalities of attention and appraisal may help facilitate interoceptive regulation in clinical populations.

## Author Contributions

VJ was involved in the conception, writing, and critical review. JD-M was involved in the conception and critical review. All authors contributed to the article and approved the submitted version.

## Conflict of Interest

The authors declare that the research was conducted in the absence of any commercial or financial relationships that could be construed as a potential conflict of interest.

## Publisher’s Note

All claims expressed in this article are solely those of the authors and do not necessarily represent those of their affiliated organizations, or those of the publisher, the editors and the reviewers. Any product that may be evaluated in this article, or claim that may be made by its manufacturer, is not guaranteed or endorsed by the publisher.
